# Structural Evolution of Silicon Nitride Anodes during Electrochemical Lithiation

**DOI:** 10.1021/acselectrochem.4c00230

**Published:** 2025-02-28

**Authors:** Adam J. Lovett, Máté Füredi, Liam Bird, Samia Said, Brandon Frost, Paul R. Shearing, Stefan Guldin, Thomas S. Miller

**Affiliations:** † Electrochemical Innovation Lab, Department of Chemical Engineering, 4919 University College London, Torrington Place, London WC1E 7JE, United Kingdom; ‡ The Faraday Institution, Quad One, Didcot OX11 0RA, United Kingdom; § Semilab Co. Ltd., Prielle Kornélia u. 2, Budapest H-1117, Hungary; ∥ Department of Engineering Science, 6396 University of Oxford, Parks Road, Oxford OX1 3PJ, United Kingdom; # Technical University of Munich, Department of Life Science Engineering, Gregor-Mendel-Straße 4, 85354 Freising, Germany; ∇ TUMCREATE, 1 CREATE Way, #10-02 CREATE Tower, 138602, Singapore

**Keywords:** operando investigation, thin film, conversion-alloying anodes, silicon anodes, electrochemical atomic force microscopy, lithium-ion battery

## Abstract

Silicon nitride (SiN_*x*_), a conversion-alloying lithium-ion battery electrode with excellent potential to replace silicon and graphite anodes, offers improved cycle stability and fast-charging capabilities. During the formation cycle(s), SiN_*x*_ irreversibly converts into a mixture of lithiated silicon and nitridosilicate matrix. However, beyond this basic understanding, there is limited fundamental insight into how the post-conversion structure results in improved electrochemical performance. This significantly hinders the optimization and commercialization prospects of SiN_*x*_ anodes. Herein, *operando* electrochemical atomic force microscopy is used to uncover the morphological and chemo-mechanical changes of SiN_*x*_ thin films during the conversion reaction. We elucidate that the post-conversion SiN_*x*_ forms silicon domains embedded within a matrix with a core-shell-like structure comprised of a stiff outer nitridosilicate surface and softer inner Si-rich core. The silicon domains that form have very stable dimensions (∼100 nm in diameter) that, crucially, remain smaller than the critical cracking threshold of silicon. This results in a more mechanically robust anode, anticipated to be free from the adverse effects of cracking, pulverization, and subsequent capacity fade. Our work marks an important advance in the fundamental understanding of silicon nitride anodes and offers a pathway to their incorporation into next-generation batteries.

## Graphical abstract

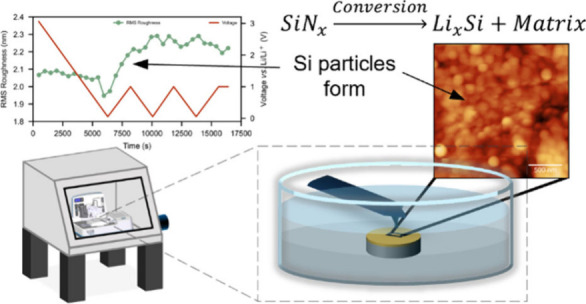

## Introduction

While silicon is increasingly being utilized as a component of commercial Li-ion batteries, owing to its high specific capacity (3579 mAh g^–1^, Li_3.75_Si), low working potential (0.4 V *vs* Li/Li^+^), abundance, low cost, and low toxicity,^1–3^ issues related to its volume expansion during lithiation (> 300% increase) mean enormous engineering effort must be employed to render silicon containing anodes stable. Without intervention, Si expansion results in fracturing and morphological changes of the anode,^4–7^ creating an unstable solid-electrolyte interphase (SEI)^7,8^ and a dynamic interface,^3^ which together drive significant capacity fading, and ultimately terminal failure mechanisms such as delamination and pulverization of the anode.^9^ For these reasons, Si is still typically utilized in a mixed graphite/silicon anode containing a small proportion (typically 10-20 wt %) of active silicon.

Recently, silicon nitride (SiN_*x*_) has gained recognition as an alternative anode active material that overcomes the significant capacity fade of Si anodes whilst retaining a high specific capacity (>1000 mAh g^–1^).^10–17^ SiN_*x*_ is known to be a conversion-alloying anode, where during the initial lithiation (formation cycles) it irreversibly converts into an intimate mixture of matrix (lithiated nitridosilicates, Li_*x*_Si_*y*_N_*z*_) and redox active components (Li_*x*_Si). This mechanism offers significantly improved cycle performance and lifetime in subsequent cycles, attributed to the matrix limiting the pulverization and fracturing of the active silicon domains, thus mitigating against the aforementioned degradation pathways.^14^ However, beyond this basic understanding, there is little fundamental evidence to confirm the intricacies of the lithiation of silicon nitride. Post-conversion, the morphology of SiN_*x*_ based anodes are commonly described as discrete domains of finely divided silicon embedded in matrix.^13–15^ The initial matrix composition has been assigned to Li_2_SiN_2_ from pair distribution functions (PDF), X-ray photoelectron spectroscopy (XPS) and ^7^Li nuclear magnetic resonance spectroscopy (NMR) measurements.^14,15^ However, while early studies proposed that the matrix is redox inactive,^12–14^ recent ^7^Li NMR studies suggest instead that the Li_2_SiN_2_ matrix does actively participate in the electrochemical reactions, reversibly converting first to Li_5_SiN_3_ and Si, and then irreversibly to Li_3_N and Si.^15^ Consequently, it has been suggested that SiN_*x*_ post-conversion is better described as an amorphous solid solution of silicon and lithiated nitridosilicates (Li_*x*_Si_*y*_N_*z*_), where only the short-range chemical environment resembles any given phase.^15^ Either way, it is clear that there is a symbiotic relationship between the silicon and matrix phases which warrants further investigation to enable material optimization.

The discrepancies in the literature are due to the key role that amorphization plays in the lithium storage mechanism of silicon nitride.^18^ This severely limits the characterization techniques that can be used to accurately study SiN_x_. Notably, diffraction-based approaches such as X-ray diffraction (XRD) and electron microscopy (EM) techniques (scanning electron microscopy, transmission electron microscopy etc.) which require crystalline material to generate a diffraction pattern cannot provide insightful information into the post-conversion structure. Indeed, where EM has been employed limited information about the fine structure of the post-lithiation SiN_*x*_ is discernable.^12–15^ Regarding the topographical relationship of silicon and the matrix phases, previous studies relied on energy-dispersive X-ray spectroscopy (EDX), which is not sensitive to lithium (and has low sensitivity to nitrogen), hence will always struggle to differentiate between phases of the same ternary phase diagram (Li–Si–N). Likewise, XPS characterization poses assignment challenges owing to a weak lithium signal plus a lack of reference binding energies available for Li_*x*_Si_*y*_N_*z*_ phases.^15^ Whilst previous PDFs and NMR studies have shown promise in identifying the matrix composition, they provide no information on their topographical distribution throughout the anode. Furthermore, reactive molecular dynamics studies identified that the delithiation of post-conversion SiN_*x*_ facilitated the separation into nitrogen-rich and Si-rich regions,^18^ however, the small size of such models (Angstrom scale) inhibits their relevance to phenomena observed at the bulk scale.

When complex materials challenges analogous to this SiN_*x*_ case have been previously faced in battery science, *operando* characterization has proven pivotal in providing answers. However, to date there are no *operando* investigations into the alloying-conversion reaction of silicon nitride, or the tracking of morphological changes of SiN_*x*_ during the formation cycles. In this endeavor, *operando* electrochemical atomic force microscopy (EC-AFM) is the ideal technique to study amorphous systems such as silicon films (a-Si),^7,8,19,20^ enabling insights into the morphological, mechanical, chemical, and physical properties of battery materials when they evolve under electrochemical control.^21^ Interestingly, AFM can not only image the surface with high precision, but can also be used to extract information on the mechanical properties of electrode surface, in addition to real time *operando* detection and mapping of morphological changes such as phase changes, particle cracking or dendritic growth.^22–24^

Herein, we use *operando* EC-AFM to elucidate the conversion reaction of silicon nitride. By using amorphous SiN_*x*_ thin films grown by pulsed laser deposition (PLD) as a model system (Figure 1a) we showcase the power of *operando* EC-AFM, in combination with *ex situ* quantitative nanomechanics (QNM) AFM, to elucidate the morphological changes during the conversion reaction and thus understand the origins of enhanced cycle performance of silicon nitride over silicon (Figure 1b). Our results reveal that the post-conversion SiN_*x*_ anode is comprised silicon domains embedded in a matrix with a core-shell-like structure, exhibiting a stiffer outer surface layer and softer inner layer ascribed to the nitrogen-rich nitridosilicate phases and Si-rich phases, respectively. The stiffer matrix constricts the volume expansion so that nanosized silicon domains are retained, aiding rate performance and crucially restricting the silicon domains so that they do not exceed the critical cracking threshold of silicon. Consequently, our results demonstrate that dimensional stabilization is an innate characteristic of silicon nitride anodes, enabling improved cycle performance and lifetime over silicon. These results affirm that silicon nitride is viable alternative to existing silicon anodes.

**Figure 1:**
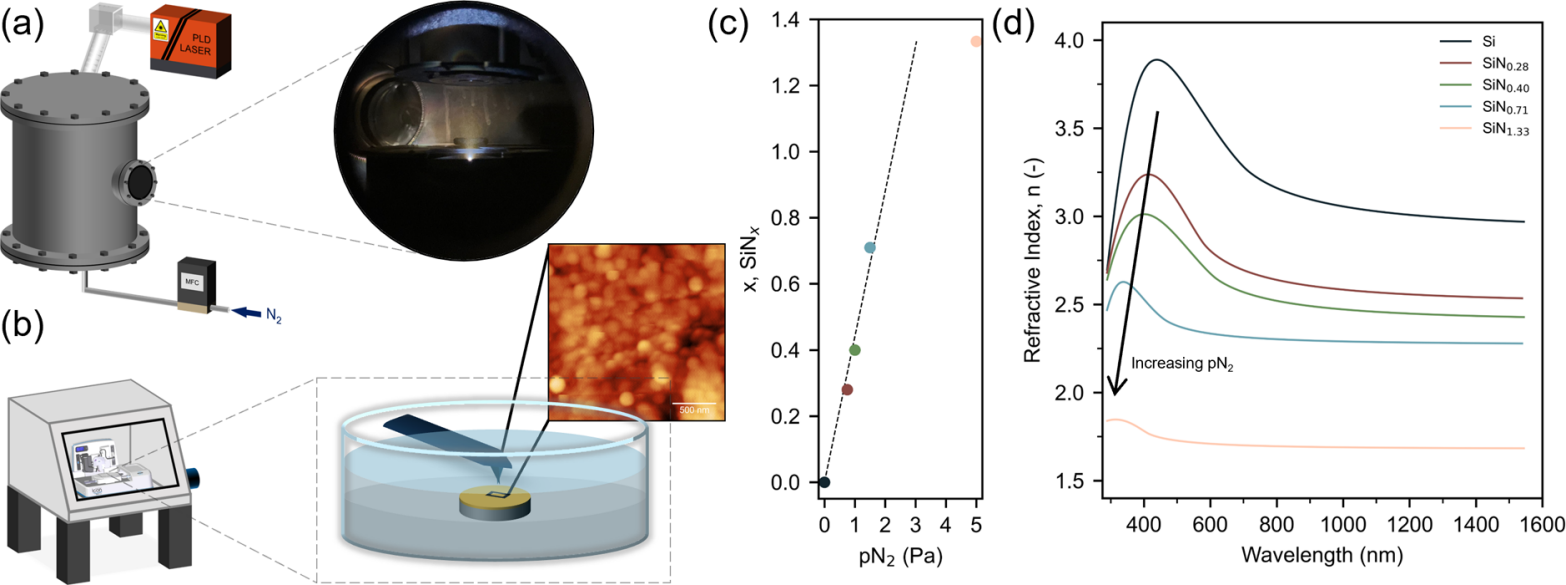
Schematics of (a) pulsed laser deposition (PLD) chamber; (b) glovebox integrated atomic force microscopy setup. (c) Composition (*x*, SiN_*x*_) dependence on pN_2_ during PLD determined from (d) ellipsometry derived refractive index data.

## Methodology

### Thin Film Growth

Silicon nitride/silicon films were grown by pulsed laser deposition (PLD) using a KrF excimer laser with a wavelength of 248 nm. Before growth, stainless steel (SS) substrates (⌀ = 15.5 mm, MTI Corp.) were cleaned with acetone in an ultrasonic bath for 5 min. Prior to deposition, the PLD chamber was evacuated to a base pressure of at least 10^–4^ Pa. Silicon films were grown under vacuum; silicon nitride films were grown between pN_2_ = 0.75-5 Pa. The target (silicon wafer, n-type undoped, MTI Corp.) was pre-ablated for 5 min with a fluence of 2.3 J cm^–2^ at 10 Hz. Films were grown with the following growth conditions: T_sub_ = 25 °C, F = 2.3 J cm^–2^, pN_2_ = 0 (silicon)/0.75-5 Pa (silicon nitride), *v* = 10 Hz, substrate-target distance = 45 mm, 1 film per growth.

### Thin Film X-ray Diffraction Characterization

Films were characterized with high resolution X-ray diffraction (XRD), performed on a Panalytical Empyrean vertical diffractometer using a Cu Kα X-ray radiation source with a wavelength of 1.5418 Å.

### Ellipsometry

Ellipsometry was performed using a Semilab SE-2000 rotating compensator spectroscopic ellipsometer. Films deposited on SS substrates were measured under ambient conditions with incident angles of 60°, 70° and 75° between 275-1600 nm wavelengths. The measured ellipsometry spectra (expressed with values Ψ and Δ for each incident angle; see Figure S1) were modelled and analyzed with the Tauc-Lorentz dispersion law using the Semilab SEA software to obtain thickness and optical dispersion (refractive index) information.

### Electrochemical Characterization

For all electrochemical characterization, silicon nitride/silicon films were transferred to an argon atmosphere glovebox (< 0.5 ppm of H_2_O and O_2_). Subsequently, cells were assembled in CR2032 coin cells (MTI Corp.) containing the test electrode (film grown on stainless steel substrate), a Celgard 2400 separator, 30 μL of electrolyte (1 M LiPF_6_ in 1:1 ethylene carbonate/diethyl carbonate (EC/DEC)) and a pre-polished lithium metal disc electrode. Electrochemical measurements were conducted on a Biologic BCS-805 potentiostat in a two-electrode setup. Galvanostatic charge–discharge cycling was conducted between 0.05-1.00 V *vs* Li/Li^+^. Rate capability testing was undertaken with the following current profile, repeated twice: 5 μA cm^–2^ (3 pre-cycles, then 10 cycles), 10 μA cm^–2^ (10 cycles), 50 μA cm^–2^ (10 cycles) and 100 μA cm^–2^ (10 cycles). Then, long term galvanostatic cycling was performed for 120 cycles at 10 μA cm^–2^ (corresponding C-rates are reported in Table S1). Cyclic voltammetry (CV) measurements were performed with a 2-electrode setup using the same cell conditions and scan rates between 0.1-1.0 mV s^–1^ with a 10-h potential hold at the cut-off voltages. Within, specific gravimetric capacities (mAh g^–1^) are reported, using the film thicknesses determined from ellipsometry. The film density was estimated using the following equation previously used for sputtered SiN_*x*_ films:^12^

$$\rho_{film}=\rho_{Si}+\frac{x\left(\rho_{SiN_{1.31}}-\rho_{Si}\right)}{1.31}$$

where *x* = [N]/[Si], *ρ*_*film*_ is the density of the film, *ρ*_*Si*_ and ρ_*SiN*_1.31__ are the densities of amorphous silicon (2.2 g cm^–3^)^25^ and amorphous SiN_1.31_ (2.9 g cm^–3^ ^26^) respectively.

### Atomic Force Microscopy

*Ex situ* and *operando* electrochemical atomic force microscopy experiments were carried out using a Bruker Dimensions Icon with ScanAsyst housed in an Ar-filled glovebox (< 0.5 ppm of H_2_O and O_2_) with RTESPA-525 AFM tips (Sb (n) doped Si with reflective Al coating, k = 200 N m^–1^, f_0_ = 525 kHz) to characterize the film morphology. Two PeakForce tapping AFM modes were undertaken. The first, o*perando* electrochemical atomic force microscopy (EC-AFM), was performed in battery electrolyte using a specialized AFM electrochemistry cell. A full schematic of the AFM cell is reported elsewhere,^23^ but is summarized in Figure 1b. Silicon nitride/silicon films grown on stainless steel substrates were affixed to the AFM stage and secured in place with adhesive polyimide film (Kapton tape) punched with a ∼ 5 × 5 mm^2^ hole. Lithium metal was used as the counter and reference electrode, which were connected to the potentiostat circuitry with nickel wire. EC-AFM measurements were performed in 1 M LiPF_6_ in 1:1 ethylene carbonate/diethyl carbonate (EC/DEC) electrolyte. CV measurements were performed using a CH Instruments 700E Series Bipotentiostat. The AFM tip is electrically isolated from the EC-AFM cell so that no current can pass through the AFM tip. The second mode, *ex situ* AFM, was performed on silicon nitride films prior cycled in CR2032 coin cells. In preparation, the film surface was rinsed with DMC to remove any residual electrolyte/SEI/salts, followed by drying inside the glovebox for 24 h under ambient conditions. The reduced Young’s modulus was mapped using the Quantitate NanoMechanics (QNM) PeakForce tapping mode using the relative mode calibrated against silicon (001) wafter (150 GPa).^27^ Further information about the method can be found elsewhere.^23^ For sub-surface QNM AFM imaging, to clean the top surface of the sample, contact mode AFM with a high force set point of 1.5 μN was used. The area cleaned (90 μm × 90 μm) was scanned eight times to remove the top surface of the film without total removal of the film. All AFM images were analyzed using the Gwyddion software.^28^

## Results

### Characterization of Pristine Silicon Nitride Films

First the Si:N ratio of the PLD grown films were characterized with ellipsometry (Figure 1d,e; see also fitted data in Figure S1). PLD has been widely used to grow high-quality model battery thin films for exploratory science owing to its relative ease and ability to control stoichiometry.^29–35^ Specifically here, key advantages of utilizing PLD are clearly demonstrated including the ability to directly control the nitrogen content by altering the pN_2_ pressure, which is well established from previous PLD silicon nitride studies,^36–38^ and the growth of films free of hydrogen incorporation that is associated with silane-based thin-film growth methods.^18^ From XRD, the absence of reflections arising from the films verify that SiN_*x*_ films are amorphous (Figure S2), as expected from previous studies.^36,39–41^ To verify the silicon nitride film composition and demonstrate how fine-tuning of the nitrogen pressure during PLD impacts the material stoichiometry, ellipsometry was used (Figure 1d,e). The exact silicon:nitrogen (*x*) ratio was determined by^12,42^

$$x=\frac{\left[N\right]}{\left[Si\right]}=\frac{4}{3}\frac{\left(n_{Si}-n_{film}\right)}{\left(n_{film}+n_{Si}-2n_{Si3N4}\right)}$$

where *n* denotes the refractive indices of the respective material at 500 nm wavelength. Here, values of *n*_*Si*_ = 3.802 and *n*_*Si3N4*_ = 1.727 were used, as determined from ellipsometry measurements of our films grown at pN_2_ = 0 Pa (a-Si) and 5 Pa (a-Si_3_N_4_). These values are in good agreement with previous literature reports for amorphous Si/Si_3_N_4_ films,^36–38,43^ confirming that films grown at pN_2_ = 0 and 5 Pa result in amorphous silicon (a-Si) and stoichiometric silicon nitride (a-Si_3_N_4_), respectively.^36^ Below pN_2_ < 1.5 Pa, the resultant films were non-stoichiometric (SiN_*x*_, 0 < *x* < $\frac{4}{3}$ ), obeying a roughly linear relationship with pN_2_ (Figure 1c), accompanied by a shift in the peak refractive index towards lower wavelengths (Figure 1d). These changes in optical properties are also observed visually as film color changes from blue (Si) to red-brown/yellow (SiN_*x*_, 0 < *x* < $\frac{4}{3}$ ) to colourless (Si_3_N_4_) in films with consistent nominal thickness (∼30 nm). Note here, these color changes are due to films exhibiting a Fabry-Pérot resonance mode, and the color exhibited is dependent on both the film thickness and composition, in addition to the reflective properties of the substrate.^44,45^

### Galvanostatic Cycling of SiN_*x*_ Thin Films

To assess the electrochemical performance of our amorphous SiN_*x*_ thin films across a range of Si:Ni compositions, mixed galvanostatic cycling at a range of current densities (μA cm^–2^) and over long durations was undertaken (Figure 2). Differential capacity analysis of the 10^th^ cycle (Figure 2a) exhibited the characteristic silicon nitride redox signatures on discharge and charge (herein labelled with prefixes X#D and X#C respectively, where X = Si for silicon or M for matrix). All films displayed the reversible silicon redox peaks between 0.05–0.50 V: Si#C1/Si#D1 corresponding to the (de)­lithiation of a-Si ⇌ Li_2.0_Si, and Si#C2/Si#D2 related to the further (de)­lithiation of a-Li_2.0_Si ⇌ a-Li_3.5_Si^46^ (for a cut-off voltage of 0.05 V *vs* Li/Li^+^_,_ chosen as it largely avoids the formation of crystalline c-Li_3.75_Si^13,47^ ). An additional redox peak, M#D1 between 0.3–0.6 V is observed for all nitrogen containing films (*x* > 0), which is not present in our silicon film (Figure 2a, dark blue). Also, the M#D1 redox peak is accompanied by a broadening of the silicon delithiation redox peaks (Si#C1, Si#C2) above 0.6 V. These features, ascribed to the lithiation of the matrix phase, gradually increase in magnitude with increasing nitrogen content, consistent with previous reports.^12,13^

**Figure 2:**
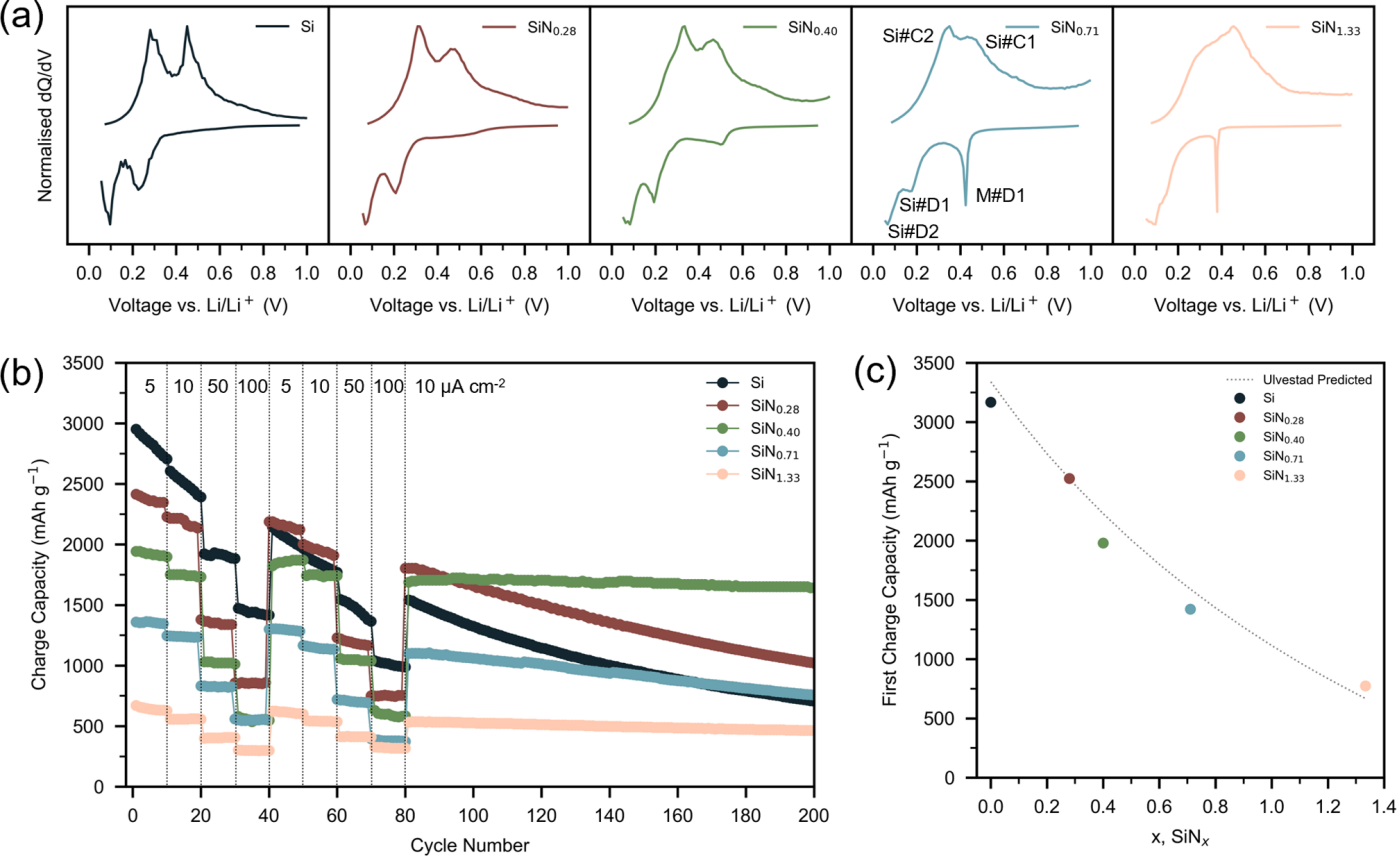
Galvanostatic cycling performance of SiN_*x*_ thin films. (a) Differential capacity analysis. All profiles show the characteristic redox peaks (labelled in SiN_0.71_ panel) of amorphous silicon between 0.05 and 0.50 V: Si#D1/Si#C1 (a-Si ⇌ a-Li_2.0_Si) and Si#D2/Si#C2 (a-Li_2.0_Si ⇌ a-Li_3.5_Si). Silicon nitride films (*x* > 0) exhibit an additional redox peak between 0.3 and 0.6 V, M#D1, ascribed to lithiation of the matrix. The magnitude of this peak increases with increasing nitrogen content. (b) Mixed rate capability and charge–discharge performance, cycled between 0.05 and 1 V *vs* Li/Li^+^. Increasing the nitrogen content decreases the charge capacity but improves the long-term cycle performance. (c) First charge capacity of our films *vs* predicted values by Ulvestad *et al*.^14^ (dashed line), which are in close agreement.

Two rounds of rate performance testing were conducted between 5–100 μA cm^–2^ for all films, before long-term galvanostatic cycling at 10 μA cm^–2^ (Figure 2b). For all films tested the measured first charge capacity correlated well with predicted values reported previously by Ulvestad *et al*.^12,14^ (Figure 2c). This further validates high-quality films and controlled Si:N stoichiometry of the films grown. The silicon film (Figure 2b, dark blue) initially displayed very high charge capacity, but showed significant capacity fade throughout the test, dropping below 1000 mAh g^–1^ after 140 cycles. Increasing the nitrogen content both increased the rate capability stabilization and the long-term cycle stability. The latter is particularly evident with long-term galvanostatic cycling (at 10 μA cm^–2^ cycle 80 onwards in Figure 2b), where limited capacity fade is evident for high nitrogen content silicon nitride films *vs* the silicon film. However, increasing nitrogen content is also accompanied by a decrease in the overall specific charge capacity, consistent with a reduction in the amount of redox active Si generated *in situ* during the forming cycles. Optimal electrochemical performance is observed for our SiN_0.40_ films (Figure 2b, green), where both high capacity and significantly reduced capacity fade are retained. This is in agreement with previous studies which typically find the best performance for SiN_*x*_ compositions between 0.3 < x < 0.9.^10,12–15^ However, we note that differing structures (nanoparticles *vs* thin films) produced with different synthetic approaches, potential impurities such as hydrogen from silane precursors,^12–14^ gradient nanoparticle structures with N-rich surfaces and Si-rich cores^10,15^ and nano-electrochemistry effects^4,29,48^ may result in discrepancies in the optimal nitrogen content. Nonetheless, all films (Figure 2) displayed a higher charge capacity than the theoretical capacity of graphite (372 mAh g^–1^) over 200 cycles,^49^ the most conventional Li-ion battery anode material.

### Cyclic Voltammetry Studies

CV measurements were also conducted to further study the redox processes and lithium diffusion dynamics of Si and SiN_0.40_ films (Figure 3). First, to correlate the electrochemical processes observed in EC-AFM measurements (see *Operando* EC-AFM Section), three CV cycles at 0.1 mV s^–1^ were performed (Figure 3a,b). Particular attention is directed towards differences between the first and second cycle for both films.

**Figure 3:**
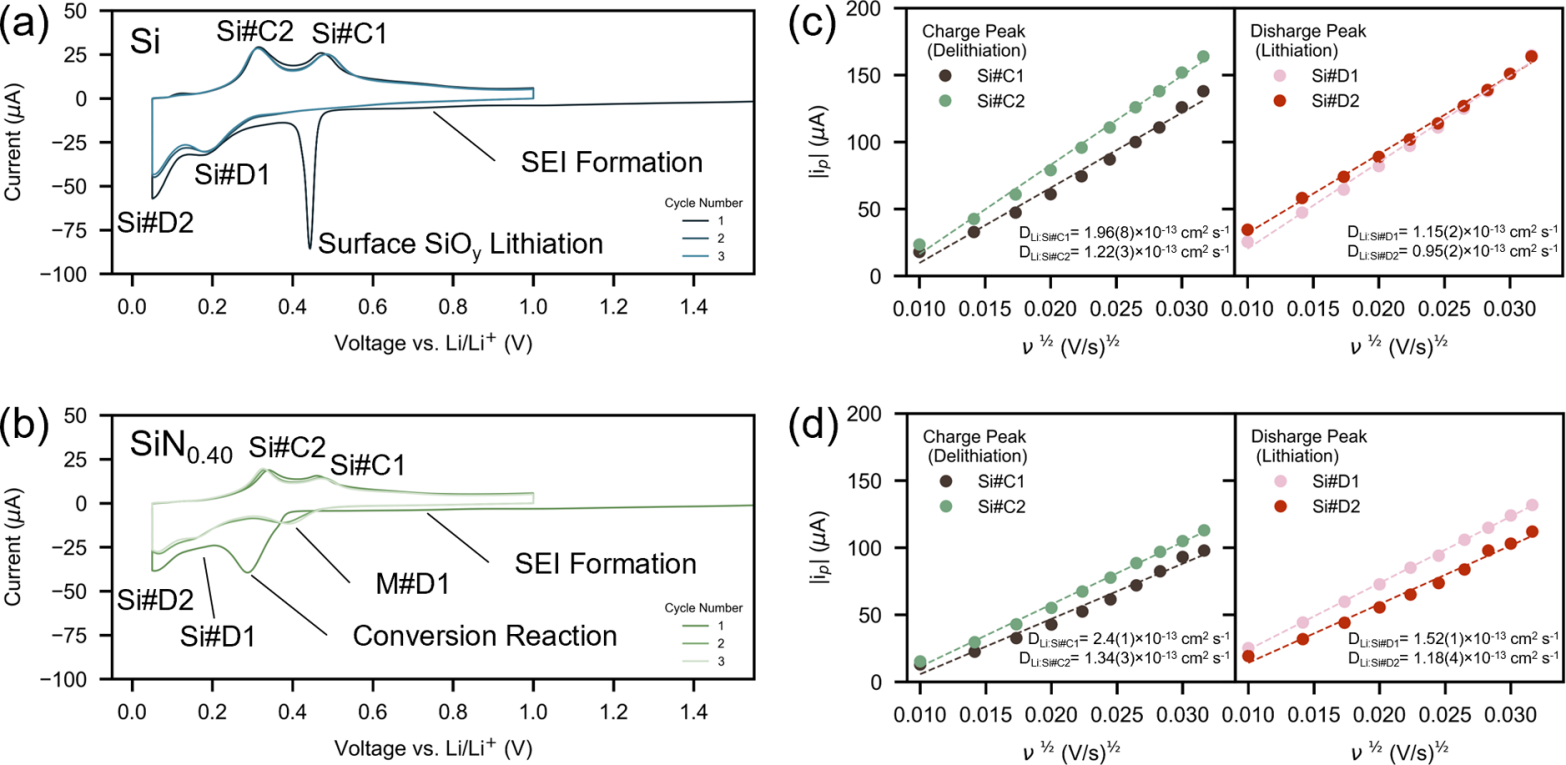
Cyclic voltammograms for (a) Si and (b) SiN_0.40_ films for the first 3 cycles at 0.1 mV s^–1^. Both films exhibit the characteristic redox peaks of a-Si: Si#D1/Si#C1 (a-Li_2.0_Si ⇌ a-Si) and Si#D2/Si#C2 (a-Li_3.5_Si ⇌ a-Li_2.0_Si). A broad redox peak starting around ∼0.8 V is present in both films, corresponding to SEI formation. The Si film contains an extra redox peak in the first cycle assigned to the irreversible lithiation of native SiO_*y*_ surface layers. The SiN_0.40_ film exhibits an additional redox peak in the 1^st^ cycle ascribed to the irreversible conversion reaction and in 2^nd^ and 3^rd^ cycles ascribed to lithiation of the matrix (M#D1). Plots of *i*_p_ *vs* ν^0.5^ for Si (c) and SiN_0.40_ (d) films. All plots are linear, confirming Randles-Sevcik equation is obeyed. Diffusions coefficients (inset) are of the order 10^–13^ S cm^–1^ for both a-Si related redox peaks (Si#C1/Si#D1 and Si#C2/Si#D2).

For the silicon film (Figure 3a), during the initial discharge (lithiation) the first feature present is a broad irreversible redox peak beginning at ∼0.8 V *vs* Li/Li^+^, corresponding to SEI formation due to the decomposition of the organic EC and DEC solvents within the electrolyte.^50,51^ This is followed by a sharp redox peak present at ∼0.44 V, which only occurs in the first cycle, ascribed to the lithiation of the native SiO_*y*_ surface layer on the silicon film.^50,52^ The exact location of this peak varies depending on the Si:O ratio of the native layer,^52^ and is hard to eliminate entirely, even in high-vacuum environments.^53^ Then, the typical reversible redox features of a-Si are observed; two broad peaks at ∼0.20 V and ∼0.06 V assigned to the characteristic lithiation of a-Si: a-Si ⇌ Li_2.0_Si (Si#D1) and a-Li_2.0_Si ⇌ a-Li_3.5_Si (Si#D2) respectively. Their charge (delithiation) counterparts are observed at 0.30 V (Si#C2) and 0.48 V (Si#C1) respectively. In subsequent cycles only the Si#C1/Si#D1 and Si#C2/Si#D2 redox peaks are present.

We now focus our attention on the SiN_0.40_ film (Figure 3b). Again, the first feature present during the initial discharge is a very broad peak beginning at ∼0.8 V *vs* Li/Li^+^, ascribed to the formation of the SEI.^50,51^ Also, as expected, the typical redox features of a-Si are present: Si#D1/Si#C1 at ∼0.15/0.46 V (a-Li_2.0_Si ⇌ a-Si) and Si#D2/Si#C2 at ∼0.06/0.34 V (a-Li_3.5_Si ⇌ a-Li_2.0_Si) respectively. An additional redox peak is present in all three cycles which requires careful attention, particularly for the first cycle which has not been previously described in detail.^12–14^ In the first discharge (lithiation), the additional peak is present at a lower voltage (∼0.29 V), before gradually shifting to higher voltages in the second (∼0.37 V) and third (∼0.39 V) cycles. Post formation (cycle 2 and 3) this additional redox peak is assigned to the lithiation of the matrix (M#D1), with the shift to higher voltages having been previously observed and ascribed to improved kinetics in the developed phase-separated SiN_*x*_ anode structure.^12,13^ Nonetheless, as we will later show in *operando* EC-AFM measurements (Figure 4 and 5), the first cycle redox peak corresponds to a significant structural change in the SiN_*x*_ anode. Hence, we propose that the first cycle lithiation peak should instead be ascribed to the irreversible conversion reaction of the SiN_*x*_ anode. Therefore, although present within a similar voltage range *vs* Li/Li^+^, it is distinctly different to the conventionally ascribed matrix lithiation peak (M#D1) which occurs at ∼0.3-0.6 V from the second cycle onwards (see Discussion).

**Figure 4:**
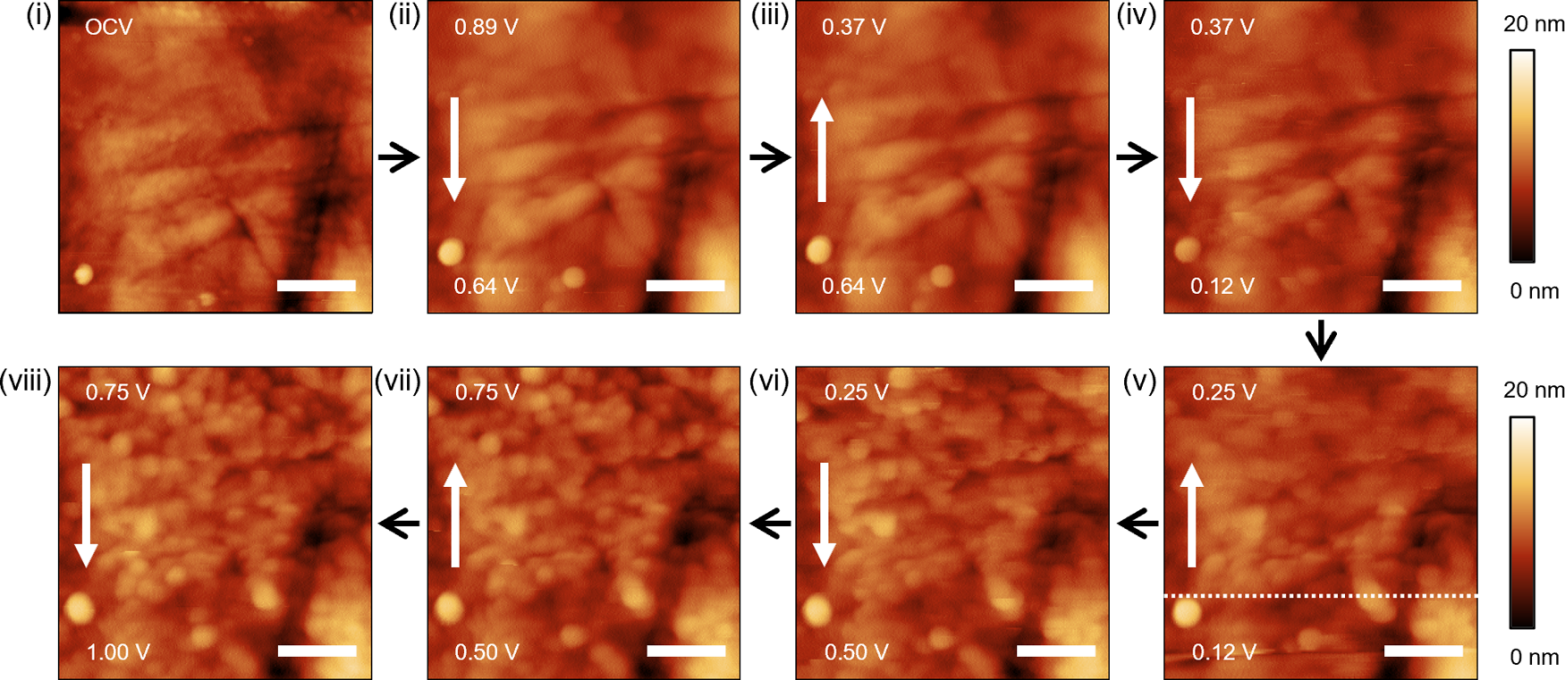
*Operando* EC-AFM images of a SiN_0.40_ film at OCV and during the first discharge–charge cycle whilst undergoing the conversion reaction between 1 V → 0.05 V → 1 V *vs* Li/Li^+^. Each image captures ∼0.25 V, with the white arrow denoting the AFM scan direction and the dashed line correspond to 0.05 V (lower cut off voltage). The clear formation of silicon domains is observed throughout the cycle, onset at *V* *<* ∼0.25 V. Scale bar = 500 nm.

**Figure 5:**
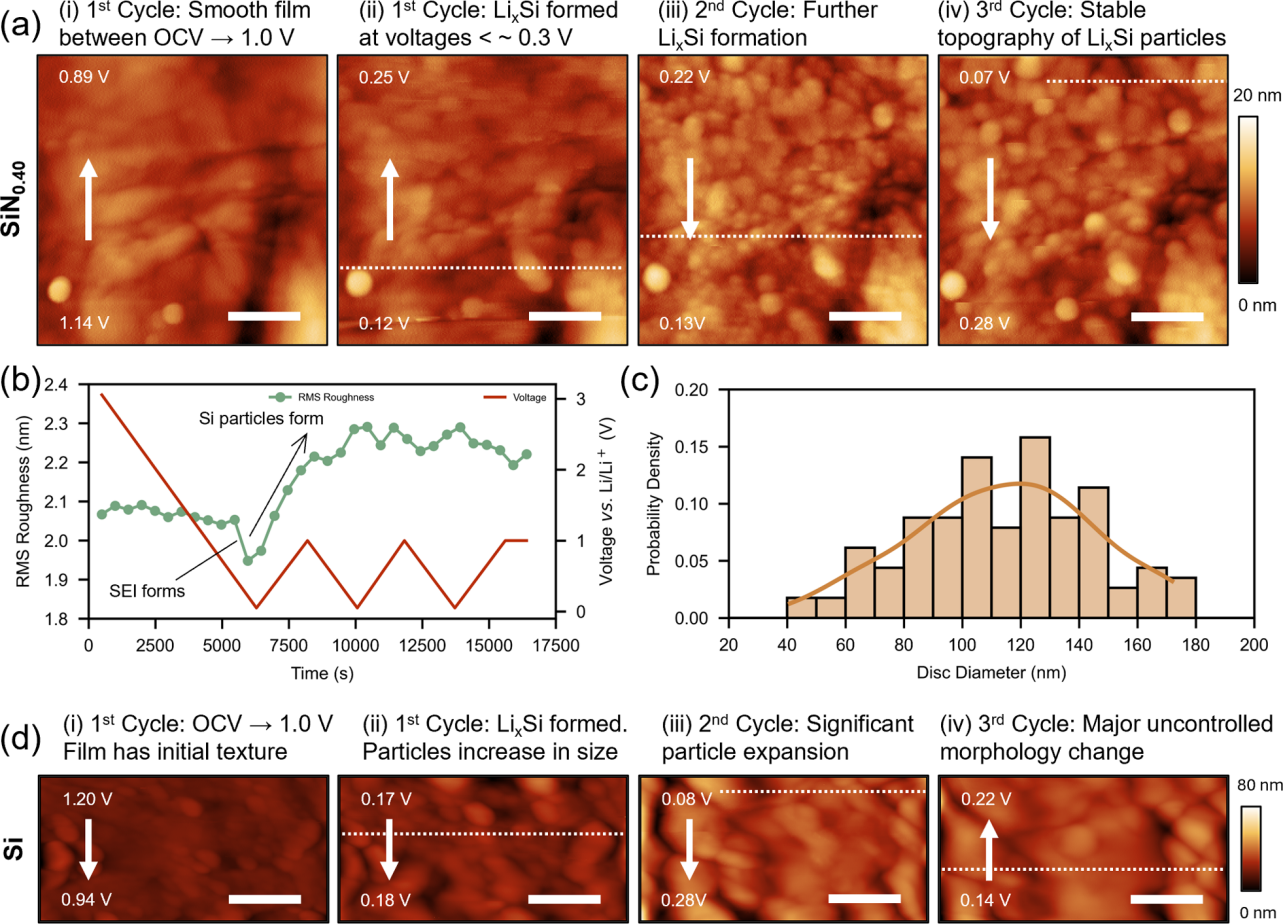
(a) *Operando* EC-AFM images of SiN_0.40_ film around 1 V (i) and 0.05 V (white dashed line) for the 1^st^ (ii), 2^nd^ (iii), and 3^rd^ (iv) cycles. The formation of silicon domains is clearly seen, and the surface topography remains very stable after 3 cycles. All scale bars = 500 nm. (b) Evolution of RMS roughness of the SiN_0.40_ film as a function of voltage and time. A clear change in RMS roughness is observed, first a reduction during SEI formation and later an increase during the onset of silicon domain formation. (c) Particle size distribution of SiN_0.40_ film in the 3^rd^ cycle. The average particle dimension is 114 ± 3 nm, crucially below the cracking threshold of silicon (∼150 nm).^4^ See Figure S5 for methodology. (d) *Operando* EC-AFM of a silicon film for comparison. The surface topography is very dynamic during cycling, with domains formed much larger than the silicon cracking threshold. All scale bars = 500 nm.

Next, we investigate the lithium diffusion dynamics of the Si and SiN_0.40_ films to assess the impact of nitrogen inclusion (Figure 3c,d). Again, both films show the typical redox features of a-Si: Si#D1/Si#C1 (a-Li_2.0_Si ⇌ a-Si) and Si#D2/Si#C2 (a-Li_3.5_Si ⇌ a-Li_2.0_Si) when collected at CV sweep rates between 0.1–1.0 mV s^–1^ (Figure S3). As also seen, the silicon nitride film voltammograms contain the additional matrix redox peak at ∼0.3–0.4 V (M#D1), which is not present in the silicon film. The diffusion coefficients for the silicon redox features are assessed using the Randles-Sevcik equation:

$$i_{p}=2.69\times 10^{5}n^{3/2}AD^{1/2}\left[C\right]\nu^{1/2}$$

at 25 °C, where *i*_*p*_ is the peak current, *n* is the number of electrons transferred (taken to be 1), *A* is the electrode area (taken to be the SS substrate area = 1.87 cm^2^), [*C*] is the bulk concentration of lithium in the electrode and ν is the scan rate. To calculate the diffusion coefficients for SiN_*x*_ films, the amount of active silicon generated *in situ* needs to be considered. To account for this, a scale factor is applied to the concentration:

$$[C]=[C_{{Li}_{3.5}Si}]\;P_{Si}\kappa$$

where [C_Li3.5Si_] is the bulk concentration of lithium in Li_3.5_Si (0.0875 mol cm^–3^) and κ is the percentage lithiation conversion factor accounting for the two redox peaks for silicon (κ = 4/7 for a-Si ⇌ a-Li_2.0_Si (Si#C1/Si#D1), and κ = 3/7 for a-Li_2.0_Si ⇌ a-Li_3.5_Si (Si#C2/Si#D2)). P_Si_ is the percentage of active silicon generated during the conversion reaction, estimated by:

$$P_{Si}=\frac{C_{g,SiN_{x}}}{C_{g,Si}}$$

where C_g,SiNx_ and C_g,Si_ are the respective first cycle theoretical reversible capacities of SiN_*x*_ and Si predicted by Ulvestad *et al*.^12–14^ This calculation assumes that during the first cycle the conversion reactions goes to completion; this is a reasonable assumption as a 10-h potential hold was utilized post sweep at 0.05 V.

Plots of *i*_*p*_ versus ν^0.5^, where *i*_*p*_ is the peak current, are linear confirming that the Randles-Sevcik equation is obeyed. For both Si and SiN_*x*_ films, diffusion coefficients for Si#C1/Si#D1 and Si#C2/Si#D2 of the order 10^–13^ cm^2^ s^–1^ (Figure 3c,d inset) are observed for both redox peaks, within the typical range reported for a-Si determined from CV.^40,54,55^ The similarity of these diffusion coefficients suggests that the incorporation of nitrogen into the silicon nitride anode does not significantly impact the silicon-based redox processes. In other words, the enhanced rate performance reported for silicon nitride is structural in origin (see Discussion).

Randles-Sevcik analysis was not performed for the M#D1 matrix peak. Several ambiguities remain regarding information required for its calculation (namely structural information required to calculate the bulk concentration of reversible lithium in the matrix).

### *Operando* EC-AFM Investigations

To investigate the morphological changes during the silicon nitride conversion reaction, *operando* EC-AFM was conducted on a SiN_0.40_ film (Figure 4 and Figure 5, electrochemistry data presented in Figure S4). This composition was chosen owing to its previously demonstrated high capacity and stable cycling performance (Figure 2). Figure 4 showcases the morphological changes during the conversion reaction (1^st^ cycle, formation). Additionally, Figure 5a displays AFM height images from four potential windows: the pre-conversion reaction surface around 1.0 V (Figure 5ai) and the post-conversion reaction surface around 0.05 V (white dashed lines) for the 1^st^ (Figure 5aii), 2^nd^ (Figure 5aiii) and 3^rd^ (Figure 5aiv) cycles, respectively. Each image captures a 0.25 V window (497 s per image, scan rate = 0.515 Hz, 256 lines). Videos detailing *operando* EC-AFM observations of the conversion and alloying reactions are reported in Supporting Media 1 (SiN_0.40_) and Media 2 (Si).

Initially, at the open current voltage (OCV) the SiN_0.40_ film displays a flat and uniform surface with minimal features present (Figure 4i). The film surface remains relatively unchanged during discharge (lithiation) from OCV (∼3.2 V) to ∼1.0 V (Figure 4ii), consistent with the absence of redox peaks in this voltage range (Figure 3b). By ∼0.8 V (Figure 4ii), the SEI has formed on the film surface (Figure 3b), coinciding with a decrease in the root-mean-squared (RMS) roughness of the film (Figure 5b), an observation consistent with previous EC-AFM studies on silicon films.^19,20^ Topographically, this presents as a subtle obscuring and broadening of the film features that are particularly noticeable when comparing images taken at OCV (Figure 4i) and around 0.8 V (Figure 4ii). Yet crucially, distinct topographical features of the film surface can still be recognized. Below 0.3 V the gradual formation of distinctly round particulate domains is witnessed (Figure 4iv), attributed to the formation of silicon nanoparticles during the conversion reaction. This structural change coincides with the first charge redox peak observed at ∼0.30 V in our previously discussed CV measurements (Figure 3b). These domains appear to further grow while the voltage remains below 0.1 V (Figure 4v), even after the reversal of the voltage sweep direction (Figure 4v white dashed line, 0.05 V) and during the first charge (delithiation) (Figure 4vi-vii).

By the second cycle, the prominent round domains are well established and observed throughout the whole film area (Figure 5aiii), marked by a clear gradual increase in the RMS roughness of the film during cycling (Figure 5b, arrow). The film topography then remained very stable into the 3^rd^ cycle (Figure 5aiv). It should be noted that after the first cycle the silicon domains remained round, and crucially, stable in size with minimal lateral volume expansion evident (further emphasized by the stable RMS roughness, Figure 5b). In addition, the peak-to-peak height (Z scale) remained stable throughout the EC-AFM measurement, strongly suggesting minimal vertical volume expansion during cycling. The distribution of particle sizes in the 3^rd^ cycle is displayed in Figure 5c, with the average disc diameter determined to be 114 ± 3 nm (the diameter of a circle with an area equal to the area of the domain). See Figure S5 for methodology. Crucially, the determined average disc diameter (114 ± 3 nm) is below the reported cracking threshold of silicon (∼150 nm),^4^ enabling aversion of silicon particle fracture during cycling and thus improving the long-term cycle performance of the SiN_0.40_ anode (see Discussion). Post-measurement, we performed additional AFM topography measurements which confirm that the region probed during EC-AFM is representative (Figure S6).

For comparison, EC-AFM was also conducted on a silicon film (Figure 5d), showing drastic differences from the SiN_0.40_ film (Figure 5a). First, the silicon film exhibited continuous morphological changes throughout the 3 cycles. The silicon domains were not uniform in shape and demonstrated drastic lateral volume expansion during cycling, consistent with previous EC-AFM studies on silicon films.^8,19,20^ Furthermore, the peak-to-peak height (Z scale) increased (from 29 nm Figure 5di, to 60 nm Figure 5div), indicating significant vertical volume expansion occurs for the silicon film (> 107%, in line with prior reports^7,8^ ). All silicon domains expanded and collided upon lithiation, resulting in a constantly evolving dynamic surface topography, almost unrecognizable from the initial surface at OCV (Supporting Media 2). Critically, by the 3^rd^ cycle (Figure 5div), the domains present were significantly larger than the cracking threshold of silicon.^4^

We note here that, as with previous EC-AFM studies, the formation of SEI does not hinder the ability to track the surface topography of the film.^19,20^ The reported SEI thickness for silicon is wide ranging, from <1 nm to >500 nm^7,56–59^ and is known to very dependent on the cycling conditions. Namely, the number of cycles, cycling rate, occurrence of fracturing/pulverization and presence (or absence) of additives in the electrolyte such as fluoroethylene carbonate (FEC)^60^ have all been shown to influence SEI thickness. Slower cycling rates (< C/2) and inclusion of long potential holds will result in thicker SEI.^59^ In these EC-AFM measurements (Figure 4 and 5), a relatively fast sweep rate is used (0.5 mV s^–1^ = 1.8 V h^–1^, ∼ 2C). Previous studies using similar rates report thin SEI formation on silicon (< 1 nm^56,57^ ), including previous EC-AFM studies on silicon films.^19,20^ This is further validated in our EC-AFM studies by the survival of film features (e.g., boundaries) during *operando* measurement (Figure 5a,d). Additionally, we avoid scanning the edges of the film which are prone to shear stress fracture events and potential delamination.^7^

### *Ex Situ* QNM AFM Investigations

To elucidate the distribution of silicon and matrix, the mechanical properties of SiN_0.40_ film surfaces were mapped (Figure 6). Here we measure the reduced Young’s modulus calculated according to the Derjaguin–Muller–Toporov (DMT) model (see Refs. 22,23 ). Three surfaces were chosen for investigation: a fresh film submerged in electrolyte (1M LiPF_6_ in 1:1 EC:DEC) at OCV (∼3.2 V) (Figure 6a); a film which was cycled 20× in a coin cell (CV, 0.1 mV s^–1^, Figure 6b) followed by cell disassembly (at 1 V, delithiated state, including cleaning with DMC to remove the SEI layer^61^ so that the SiN_*x*_ surface can be mapped absent of SEI) and *ex situ* AFM modulus mapping before (Figure 6c) and after (Figure 6d) a contact mode clean to remove the surface layers. This clean involved using a high peak force set point to scrape away the top surface of the film, proof of which is shown in Figures S7 and S8.

**Figure 6:**
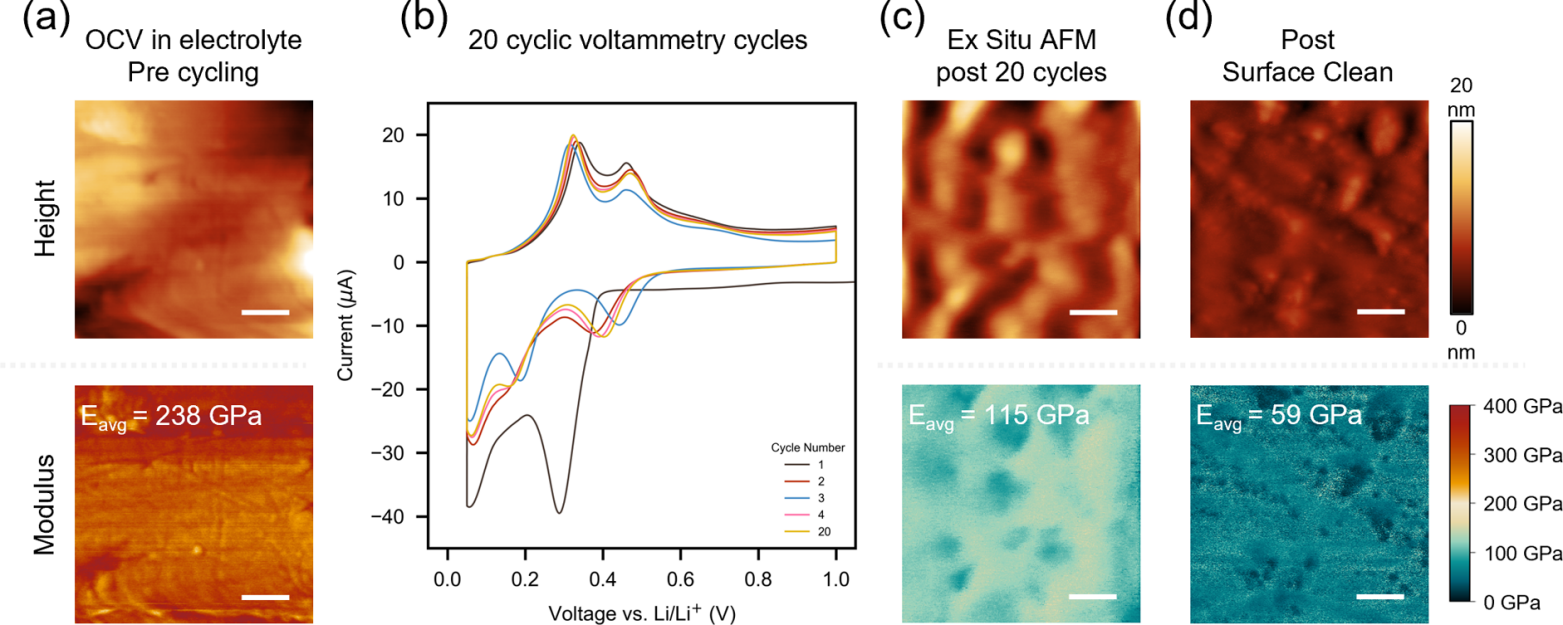
QNM AFM images mapping the surface reduced Young’s Modulus of SiN_0.40_ films. (a) At OCV in electrolyte. (b–d) After 20 CV cycles. (b) *Ex situ* (post cell disassembly, including wash in DMC to remove any SEI) plus before (c) and after (d) a high-force contact mode clean. Topographical features are similar to EC-AFM observations. Initially, the film is smooth with a high average reduced Young’s modulus (*E*_avg_ = 238 GPa). Post-cycling (c), round silicon domains are observed. Two regions are present, one characterized by a higher reduced Young’s modulus (*E* = ∼130–140 GPa) and a second with a lower reduced Young’s modulus (*E* = ∼80–90 GPa). During the high-peak force contact mode clean, the top surface is removed, leaving behind a lower reduced Young’s modulus surface (*E*_avg_ = 59 GPa). All scale bars = 200 nm.

At OCV before the SEI has formed, the SiN_0.40_ film in 1 M LiPF_6_ EC:DEC (1:1) electrolyte (Figure 6a) has a very smooth surface with minimal features present and a high surface reduced Young’s modulus (*E*_avg_ = 238 GPa). As expected for a non-stoichiometric SiN_*x*_ film, the average surface modulus sits between the reported moduli for amorphous Si_3_N_4_ = ∼270 GPa^26^ and amorphous Si = ∼90 GPa^62^), exhibiting Vegard’s law-like behavior. Post cycling (Figure 6c), round particulate topographical features are observed, analogous to those seen previously in the EC-AFM measurements (Figure 4 and 5). Modulus mapping reveals a surface with two distinct regions: one characterized by a higher reduced Young’s modulus (∼130-140 GPa) and a second with a lower reduced Young’s modulus (∼80-90 GPa) localized to the round particulate domains. The average reduced Young’s modulus (*E*_avg_ = 115 GPa) is lower than the pre-cycled sample (Figure 6a), consistent with the lithiation of silicon (a-Si *E*_a‑Si_ = 90 GPa, Li_3.75_Si E_Li3.75Si_ = ∼ 42 GPa^62^) and by extension, anticipated for silicon nitride. Note the observed moduli are higher than the reported SEI reduced Young’s modulus of silicon films (< ∼2-10 GPa),^19,58,63^ confirming effective removal of the thin SEI layer observed in EC-AFM measurements (Figure 4) with DMC washing.^61^

To investigate the inner layers, a high set point force contact mode clean was undertaken to remove the outer layers of the film (evidence of which is presented in Figure S7). The resultant surface (Figure 6d) has a lower reduced Young’s modulus (*E*_avg_ = 59 GPa), confirming removal of the higher modulus surface layer. Again, two regions are present: one characterized by a higher reduced Young’s modulus (∼80-90 GPa) distributed throughout the film and a second with a lower reduced Young’s modulus (∼30-40 GPa) localized to the round particulate domains. Both regions show a sub-surface reduction in modulus, consistent with a core-shell-like structure consisting of higher-modulus nitrogen rich surface (nitridosilicate), and a softer Si-rich core underneath (see Discussion). Additional QNM AFM images of different regions are shown in Figure S8, showing concurrent results.

## Discussion

The results above present *operando* EC-AFM insights into the origins of SiN_*x*_ performance enhancement over silicon. In combination with *ex situ* QNM AFM modulus mapping, the origins of improved cycle lifetime and rate performance of SiN_*x*_ can be understood directly from our *operando* EC-AFM observations. The data presented allows the elucidation of two critical mechanisms underpinning the enhanced performance of SiN_*x*_ anodes: controlled silicon domain sizes and a core-shell-like matrix architecture.

First from EC-AFM we observe the onset formation of silicon domains at voltages < ∼0.3 V (Figure 4 and 5), where topographically, our films contain clear particulate domains distributed throughout the film. This also corresponds with the first charge (lithiation) redox peak observed in CV measurements (Figure 3b), which we ascribe to the conversion reaction based on these observations. Such silicon domains have been previously seen in post-mortem *ex situ* electron microscopy of cycled SiN_*x*_ particles.^14,15^ However, unlike previous studies that utilized SEM-EDX to differentiate between phases,^14,15^ which cannot easily distinguish between phases within the same ternary plot (Li–Si–N, further noting that lithium/nitrogen is not sensitive to standard SEM-EDX), our QNM AFM mapping allows differentiation between phases via their differing mechanical properties. This shows that, rather than being embedded within a matrix with a discrete composition as commonly reported,^12–15^ the silicon domains are embedded within a matrix material that naturally develops a core-shell-like structure comprised of a stiff-outer layer and softer inner layer. For the matrix, the stiff outer layer is ascribed to the nitrogen-rich nitridosilicate-phases (Li_*x*_Si_*y*_N_*z*_), surrounding a softer Si-rich core, assigned based on the reported reduced Young’s moduli of a-Si_3_N_4_ and a-Si (a-Si_3_N_4_ = 270 GPa^26^ > a-Si = 90 GPa^62^). Here, the stiffer nitridosilicate matrix does not inhibit delivery of lithium to the Si domains as the matrix phases have relatively high Li^+^ ionic conductivities (Li_2_SiN_2_ = 1.1 × 10^–5^ S cm^–1^ (400 K),^64^ Li_5_SiN_3_ = 4.7 × 10^–5^ S cm^–1^ (400 K),^64^ Li_3_N = 10^–4^ to 10^–3^ S cm^–1^ (400 K)^65^). This is further validated by our CV analysis (Figure 3), as the diffusion coefficients are comparable for both Si and SiN_0.4_ films (∼10^–13^ cm^2^ s^–1^) when accounting for the *in situ* generated silicon, i.e., a-Si redox processes are rate limiting. The silicon domains also show variation in reduced Young’s modulus, having a surface modulus of 80-90 GPa, and a sub-surface modulus of 30-40 GPa. Tentatively, this difference is ascribed to differing degrees of lithiation (a-Si *E*_a‑Si_ = 90 GPa, Li_3.75_Si E_Li3.75Si_ = ∼ 42 GPa^62^), but the surfaces being capped by a stiffer nitrogen-rich phase(s) cannot be ruled out unambiguously.

Our AFM measurements provide interesting findings into the post-conversion silicon nitride structure, an area where existing literature presents conflicting interpretations. Initial studies proposed that the structure consists of discrete silicon particles embedded in a Li_2_SiN_2_ matrix.^12,13^ In contrast, a later study proposed the structure is better described as an amorphous solid solution where no distinct crystalline phases are present, but rather a local chemical environment that resembles the composition of the respective thermodynamically stable phases Li_2_SiN_2_, Li_5_SiN_3_, Li_3_N, and Li_*x*_Si.^15^ The AFM measurements we present here support elements of both proposed structures. Our EC-AFM and QNM-AFM measurements support the formation of discrete silicon domains with sizes of the order of 100 nm embedded in a matrix. Also, the variation in surface and sub-surface moduli measured by QNM AFM modulus mapping support the matrix being comprised of more than one phase. This appears to be core-shell-like in nature where the outer surface is stiffer than the inner core, which could be consistent with either a discrete or a graduated structure. Interestingly, our observations are consistent with a recent atomistic modelling study that supports the favorability of phase-separation into nitrogen-rich and Si-rich regions,^18^ which would be anticipated to exhibit different mechanical behavior. Yet, further work is necessary to unequivocally confirm the nature of the matrix structure (core-shell or graduated), a non-trivial task owing to the key role amorphization plays severely limiting the characterization techniques that may be utilized to reveal useful information.

Secondly, with *operando* EC-AFM we observe the silicon domain sizes of the SiN_0.40_ film remain very stable over multiple cycles, with an average disc diameter of 114 ± 3 nm after 3 cycles (Figure 5a). This stability contrasts significantly with the silicon film, where the surface morphology is very dynamic and almost unrecognizable after each cycle, with domains >500 nm present (Figure 5d). Such dynamic interfaces are undesirable, as they lead to pulverization, unwanted side-reactions that thicken the SEI layer, and ultimately rapid deterioration of the anode capacity and cycle life.^3^ Such capacity fade is evident for our Si film (Figure 2b, dark blue), which only retains∼ 30% of its initial charge capacity after 150 cycles. Whereas, our SiN_0.40_ film (Figure 2b, green) retains 85% of its initial charge capacity after 150 cycles, with a loss of only 3.7% during extended cycle performance testing between the 80^th^ and 200^th^ cycle (post rate capability testing). Thus, the stable surface morphology of the silicon nitride anode post-conversion is highly-beneficial, and a contributing factor to improved cycle life.

Our QNM AFM measurements (Figure 6) further reveal the mechanical properties of the matrix plays a pivotal role in the improved electrochemical performance. The stiffer nitrogen-rich matrix constricts the softer silicon domains, inhibiting the volume expansion during lithiation. Consequently, this controls the silicon domain sizes that form post-conversion, as directly seen in our EC-AFM experiments (Figure 4 and 5). A key design strategy to mitigate against volume expansion challenges in silicon anodes is dimensional stabilization, that is, reducing the size of the redox active silicon particles below a critical fracturing threshold diameter (∼ < 150 nm).^4^ Improved performance is achieved by nano-sized silicon alleviating strain such that fracture events do not occur.^4^ For silicon nitride, dimensional stabilization is achieved spontaneously due to the mechanical property relationships of the matrix and silicon. The stiffer matrix phase controls the size of the silicon domains so that nanosized dimensions are formed, crucially, below the critical fracturing threshold diameter of silicon (∼ < 150 nm,^4^ SiN_0.40_ average particle size = 114 ± 3 nm after 3 cycles). This leads to a mechanically stronger anode, expected to resist cracking, pulverization, and thus the associated capacity fade. Further, by extension, the resulting SEI is more stable due to the absence of fracture.^7,8,58^ Both effects combine to result in improved long-term cycle performance, characteristic of silicon nitride anodes (Figure 2).^14^

Finally, the post-conversion topography also facilitates improved rate performance, which is widely reported for silicon nitride anodes.^12–15^ This is primarily due to two structural features. First, the retention of nano-sized domains due to matrix constriction, which are facile to (de-)­lithiate due to shortened diffusion pathlengths, helps overcome performance shortcomings due to sluggish ionic transport that results in polarization effects at the electrode interface.^29,66,67^ Secondly, the presence of the matrix phase comprised of high ionic-conducting lithiated nitrdosilicates aids delivery of lithium to the silicon domains.^12,14^ Together, these structural features create a post-conversion structure with inherent characteristics that facilitate enhanced rate performance.

## Conclusions

In summary, we have utilized *operando* EC-AFM to map the morphological changes of a SiN_0.40_ film during the alloying conversion reaction. In combination with *ex situ* QNM AFM reduced Young’s modulus mapping, we elucidate the SiN_*x*_ film forms silicon domains and a matrix with aa core-shell-like structure comprised of a stiff nitridosilicate outer layer and softer inner Si core. We further reveal that the silicon domains that form have very stable diameters during cycling (∼100 nm in diameter). In essence, the higher modulus outer nitridosilicate-matrix behaves as an active-SEI, helping buffer the volume expansion of the inner Si particles. Nano-sized dimensions are retained, aiding rate performance, and crucially restricting the silicon domains so that they do not exceed the critical cracking threshold of silicon, facilitating a more mechanically robust anode. Through elucidation of these desirable characteristics by *operando* EC-AFM, our work marks an important advance toward the fundamental understanding of silicon nitride anodes and showcases their potential as an alternative high-silicon content anode in next generation batteries.

## Supplementary Material



## Notes

Fitted ellipsometry data, table of corresponding C-rates, X-ray diffraction of selected films, variable scan rate CVs, comparative EC-AFM cell and coin cell CVs, Si domain size measurement methodology, additional AFM topography images, proof of AFM surface cleaning, additional QNM AFM images, and supporting media videos of EC-AFM (DOCX)
