# Supplementary material for: Structural Evolution of Silicon Nitride Anodes during Electrochemical Lithiation
Source: ACS Electrochem. 2025 Feb 28;1(6):962–73. doi: 10.1021/acselectrochem.4c00230 (PMC12147161; doi:10.1021/acselectrochem.4c00230)
Supplement: Supplementary file 1 [file ec4c00230_si_001.docx]

**Structural Evolution of Silicon Nitride Anodes During Electrochemical Lithiation**

Adam J. Lovett^1,2*^, Máté Füredi^1,3^, Liam Bird^2,4^, Samia Said^1^, Brandon Frost^1,2^, Paul Shearing^2,4^, Stefan Guldin^1,5,6^, Thomas Miller^1,2*^

^1^Department of Chemical Engineering, University College London, Torrington Place, London WC1E 7JE, United Kingdom

^2^The Faraday Institution, Quad One, Didcot OX11 0RA, United Kingdom

^3^Semilab Co. Ltd., Prielle Kornélia u. 2, Budapest H-1117, Hungary

^4^Department of Engineering Science, University of Oxford, Parks Road, Oxford OX1 3PJ, United Kingdom

^5^Technical University of Munich, Department of Life Science Engineering, Gregor-Mendel-Straße 4, Freising 85354, Germany

^6^TUMCREATE, 1 CREATE Way, #10-02 CREATE Tower, Singapore 138602

*Correspondences: adam.lovett@ucl.ac.uk; t.miller@ucl.ac.uk

**Table of Contents**

**Supporting Figures and TablesS2**

Figure S1: Fitted ellipsometry dataS2

Table S1: Long-term galvanostatic corresponding C-rates S2

Figure S2: X-ray diffraction patterns of selected filmsS3

Figure S3: Variable scan rate cyclic voltammogramsS3

Figure S4: Comparative EC-AFM and coin cell cyclic voltammogramsS4

Figure S5: Si domain size measurement methodologyS5

Figure S6: Additional AFM topography imagesS6

Figure S7: Proof of AFM surface cleanS6

Figure S8: Additional QNM AFM images including *in* situ cleaningS7

**Supporting References**S8

**Supporting MediaS9**

Supporting Media 1: *Operando* EC-AFM of SiN_0.40_ thin film video S9

Supporting Media 2: *Operando* EC-AFM of Si thin film video S9

**Supporting Figures and Tables**

Figure S1: Measured and fitted ellipsometry data (expressed with values Ψ and Δ for each incident angle: 75°, 70° and 60°) for (a) Si (b) SiN_0.28_ (c) SiN_0.40_ (d) SiN_0.71_ and (e) SiN_1.33_ thin films. Note, for fitting all spectra (a-e) the same SiN_x_ optical model was used.


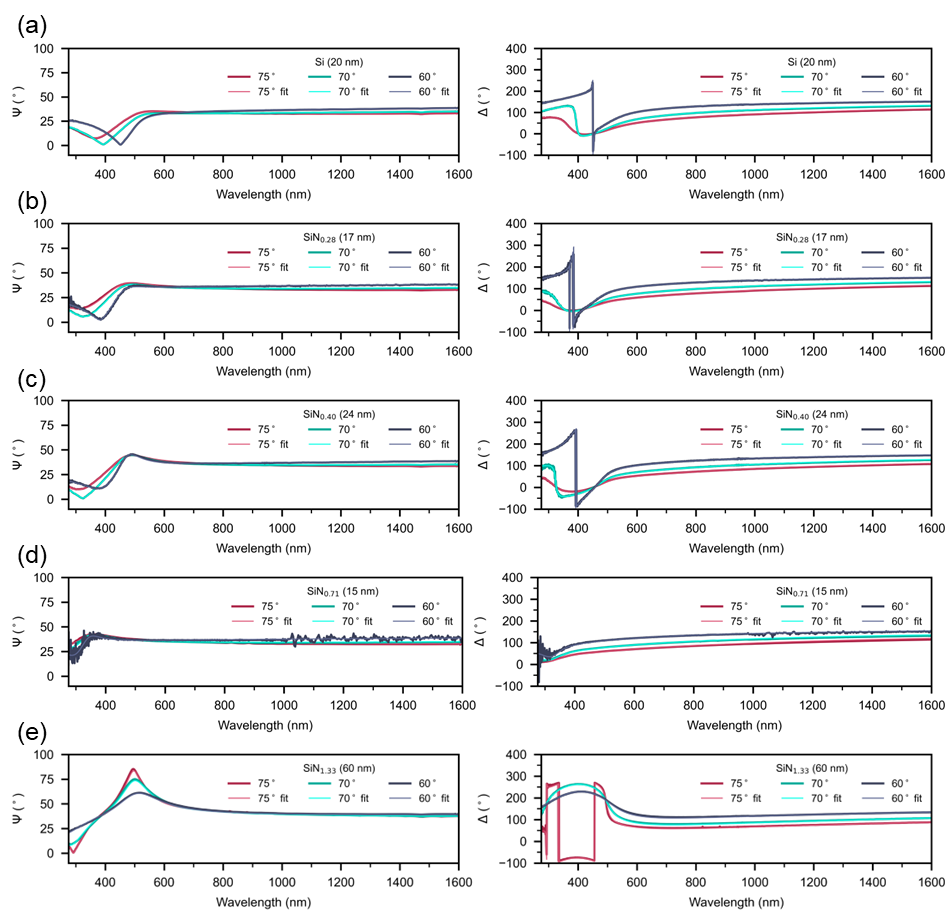


Table S1: Corresponding C-rates for long-term galvanostatic cycling performed for 120 cycles at 10 μA cm^-2^

| **x, SiN_x_** | 0 | 0.28 | 0.40 | 0.71 | 1.33 |
| --- | --- | --- | --- | --- | --- |
| **C rate @  10 µA cm^-2^** | 0.48 | 1.1 | 1.35 | 2.6 | 1.15 |


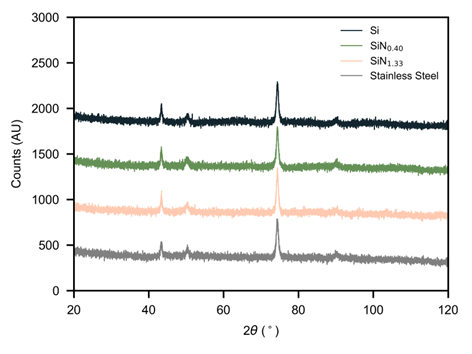


Figure S2: X-ray diffraction patterns of selected Si/SiN_x_ thin films referenced against a stainless-steel substrate. No reflections arise from the film, confirming all films are amorphous.


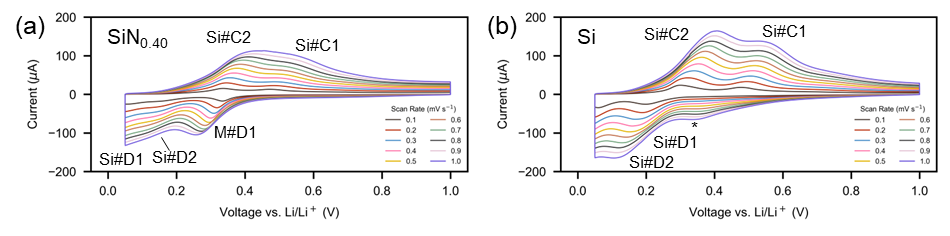


Figure S3: Cyclic voltammograms for (a) SiN_0.40_ and (b) Si thin films collected between 0.1-1.0 mV s^-1^. All redox peaks are labelled (see Cyclic Voltammetry Studies Section). Note, the additional redox peak labelled by the asterisk in the silicon film arises due to the gradual formation of a small quantity of Li_3.75_Si during extended cycling ^1^.


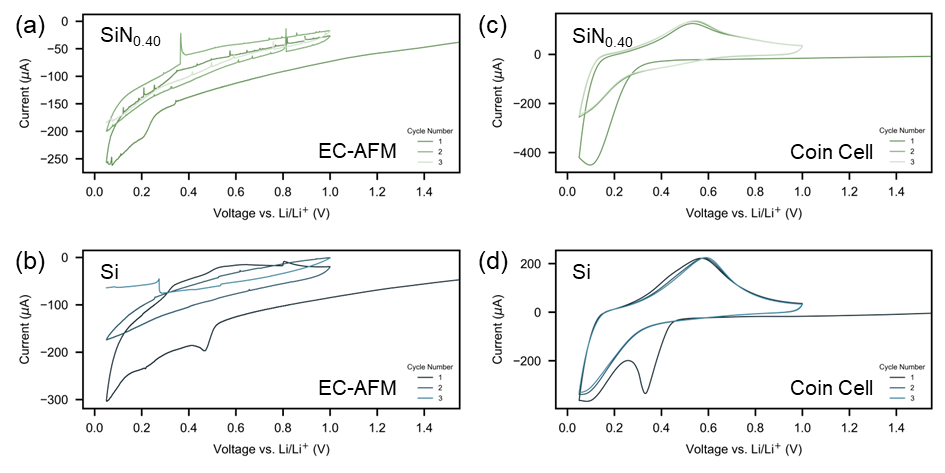


Figure S4: Cyclic voltammograms collected at 0.5 mV s^-1^ during EC-AFM (a,b) and in coin cells (c,d) under representative conditions for SiN_0.40_ and Si thin films respectively. A comparison is made between coin cell and EC-AFM CVs primarily for two reasons: 1) The EC-AFM cell uses a non-optimal volume of electrolyte (> 5 ml). This can reduce the CV signal from the material of interest due to high residual electrolyte current contributions. This is particularly the case for thin films which, by virtue of being thin, have a low mass loading of active material. 2) Due to the open-top nature of the EC-AFM cell, electrolyte evaporation is unavoidable, which consequently influences the CV features as the experiment progresses.

Figure S5: Particle size distribution analysis of a SiN_0.40_ film accompanying Figure 5c. The raw image (a) is segmented using a 2-dimensional (2D) Otsu’s method.^2^ This approach uses a “global” and “local” threshold to separate particles from the background, in addition with a salt-and-pepper noise reduction filter. The approach is well-suited to images where there is an uneven background,^3^ which is often the case in AFM images. This was followed by using the ImageJ Beat plugin particle recognition algorithms^4^ to identify particles and colour then individually (c). Then, the disc diameter of each identified particle was calculated. Close inspection of (c) reveals that some particles are poorly segmented or coalesced, which is an artefact of the segmentation process that cannot be eliminated entirely. In fact, finding a set of segmentation parameters that adequately separates all particles present is non-trivial. To overcome this, three constraints are used to remove poorly segmented particles: a size constraint (40 nm ≤ disc diameter ≤ 172 nm), an aspect ratio constraint (aspect ratio > 0.55) and a roundness constraint (roundness > 0.55). Only particles that satisfied all three constraints (114 of 223 identified particles) were included in the particle size distribution analysis in Figure 5c (average disc diameter = 114 ± 3 nm). Further, to verify the soundness of this approach, the average properties of 10 selected well-segmented particles (numbered in (c)) was calculated and found to have the following values: disc diameter = 142 nm, aspect ratio = 0.88 and roundness = 0.75. Crucially, the average disc diameter of these particles is still below the reported cracking threshold of silicon (~ 150 nm,^5^ see Discussion). Finally, it should be noted that AFM is a tip-sample convolution technique that over-estimates feature sizes. Hence, the measured particle sizes should be taken as an upper limit of their real size.


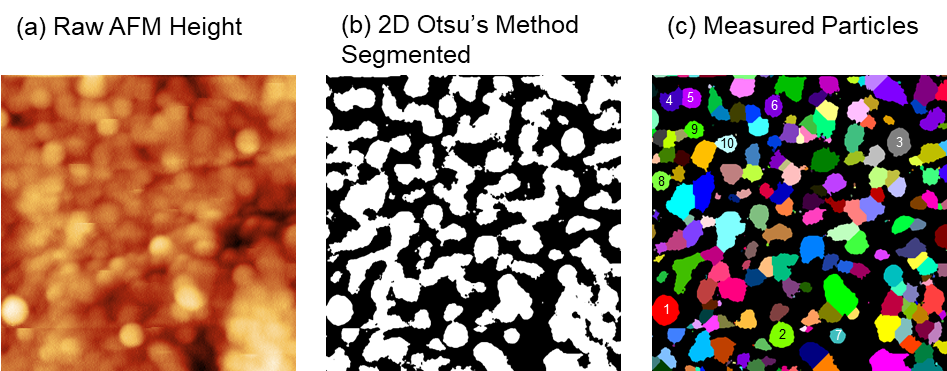

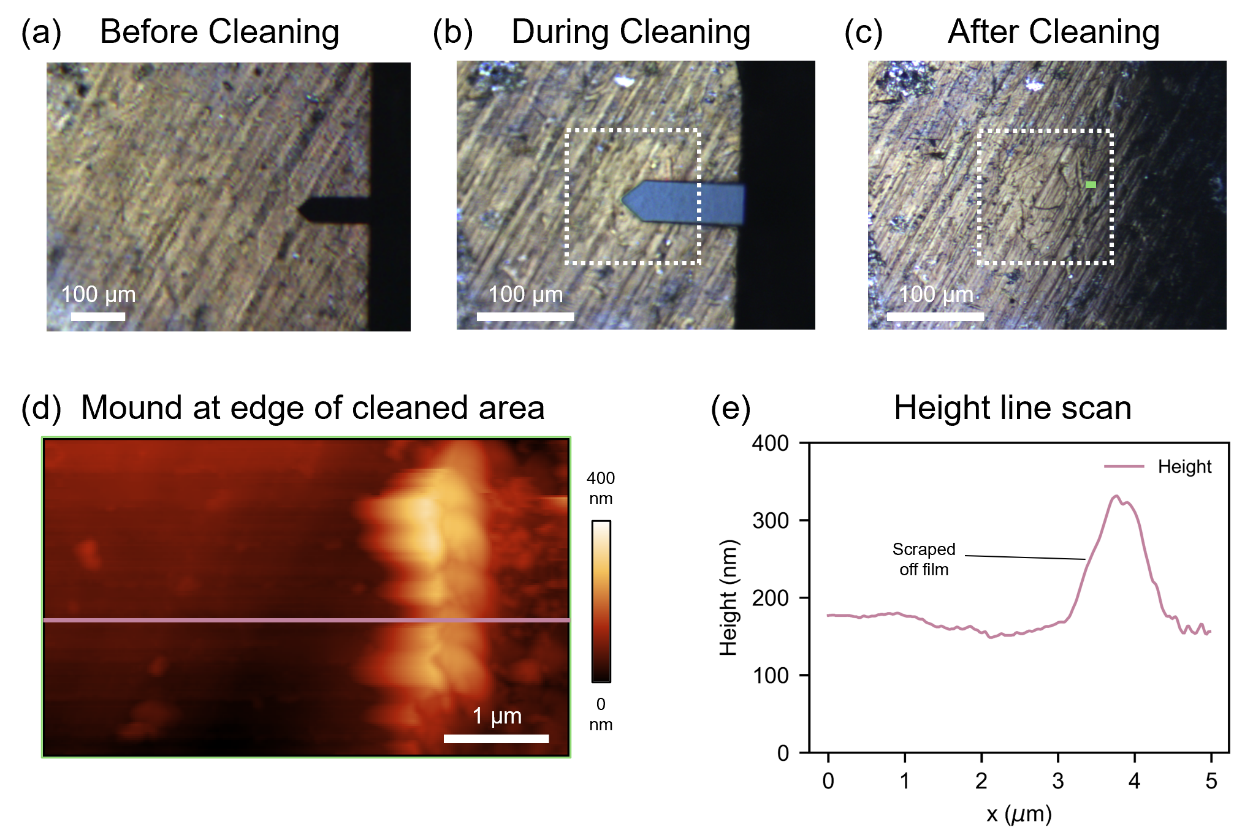


Figure S7: Proof of the removal of the outer layer of a SiN_0.40_ film. (a,b,c) Optical images captured (a) before, (b) during and (c) after the AFM cleaning process. A clear square (within the white dashed box) can be seen where the film was cleaned (90 μm × 90 μm scan area). (d) AFM height image taken from green box in (c). (e) Accompanying line scan taken from the pink line in (d). A mound with ~ 100 nm height is observed on the boundary of the cleaned area comprised of scraped off film.


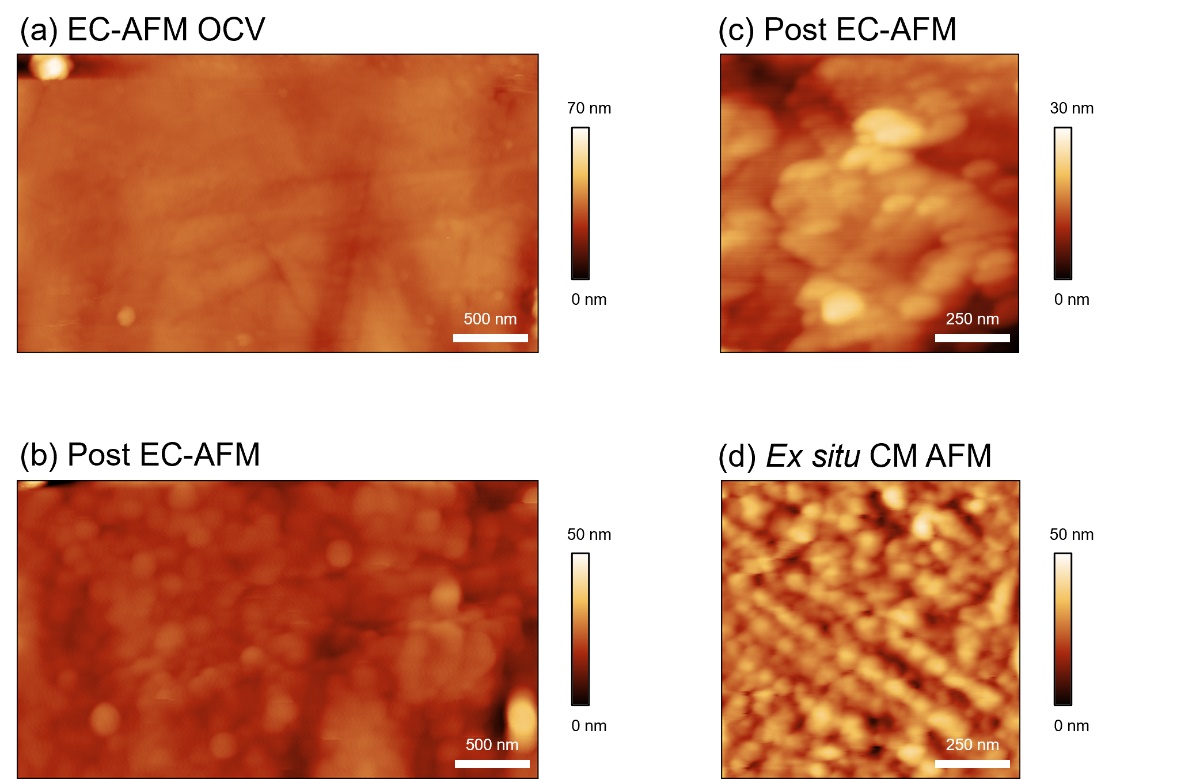


Figure S6: Additional AFM topography images of SiN_0.40_ film (a,b) EC-AFM image taken at (a) open current voltage before starting cycling and (b) post EC-AFM after cycling stopped. These are taken over a larger area than Figure 4 and 5. (c) Post EC-AFM after cycling of a different region of the film. (d) Ex Situ contact mode AFM (CM AFM) taken of the SiN_0.40_ film. All images post conversion show clear round silicon domains.


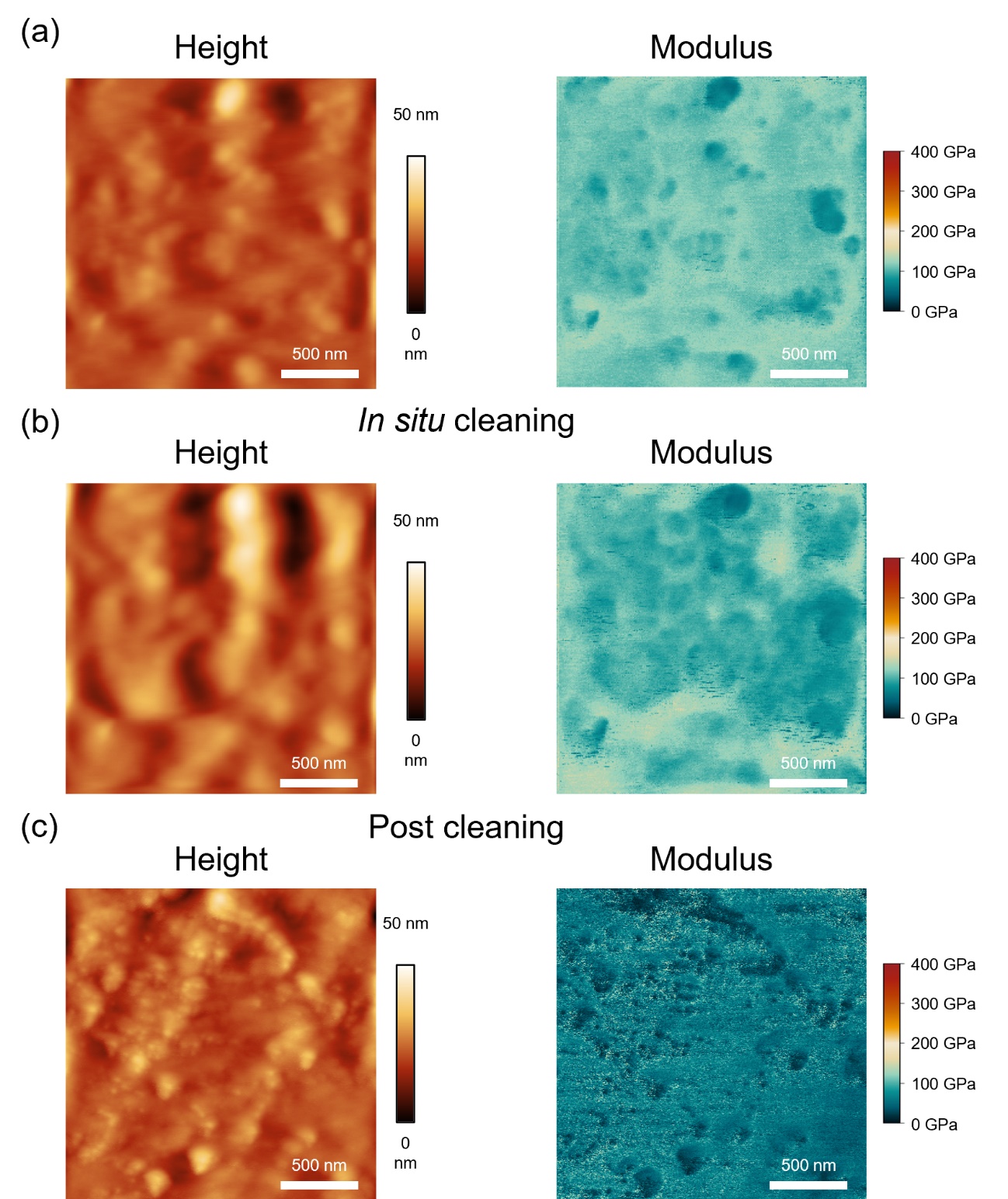


Figure S8: QNM AFM height and modulus maps (a) before cleaning and (b) in situ during high peak force tapping cleaning of the same area. From comparison of the modulus maps, it is clearly seen that surface in (b) during cleaning has a lower modulus and is consistent with the (partial) removal of the top surface. Note, this differs from the contact mode cleaning reported in Supporting Figure S7 and Figure 6c. Here, the tip is operating in a tapping mode, which is only effective at partially removing the top layer as the method intentionally minimises contact with the surface. But, the benefit is the AFM tip does not need to be replaced post cleaning, hence the same region can be imaged, as demonstrated by the height images. (c) Additional QNM AFM height and modulus image of a different area, showing concurrent results with Figure 6d.

**Supporting References**

1. Iaboni, D. S. M. & Obrovac, M. N. Li 15 Si 4 Formation in Silicon Thin Film Negative Electrodes . *J. Electrochem. Soc.* **163**, A255–A261 (2016).

2. Liu Jianzhuang, Li Wenqing & Tian Yupeng. Automatic thresholding of gray-level pictures using two-dimension Otsu method. IEEE China Int. Conf. Circuits Syst. 325–327 (1991).

3. Xing, J., Yang, P. & Qingge, L. Robust 2D Otsu’s Algorithm for Uneven Illumination Image Segmentation. *Comput. Intell. Neurosci.* **2020**, (2020).

4. Münch, B., Gasser, P., Holzer, L. & Flatt, R. FIB-nanotomography of particulate systems - Part II: Particle recognition and effect of boundary truncation. *J. Am. Ceram. Soc.* **89**, 2586–2595 (2006).

5. Liu, X. H. *et al.* Size-Dependent Fracture of Silicon. *ACS Nano* **6**, 1522–1531 (2012).

**Supporting Media**


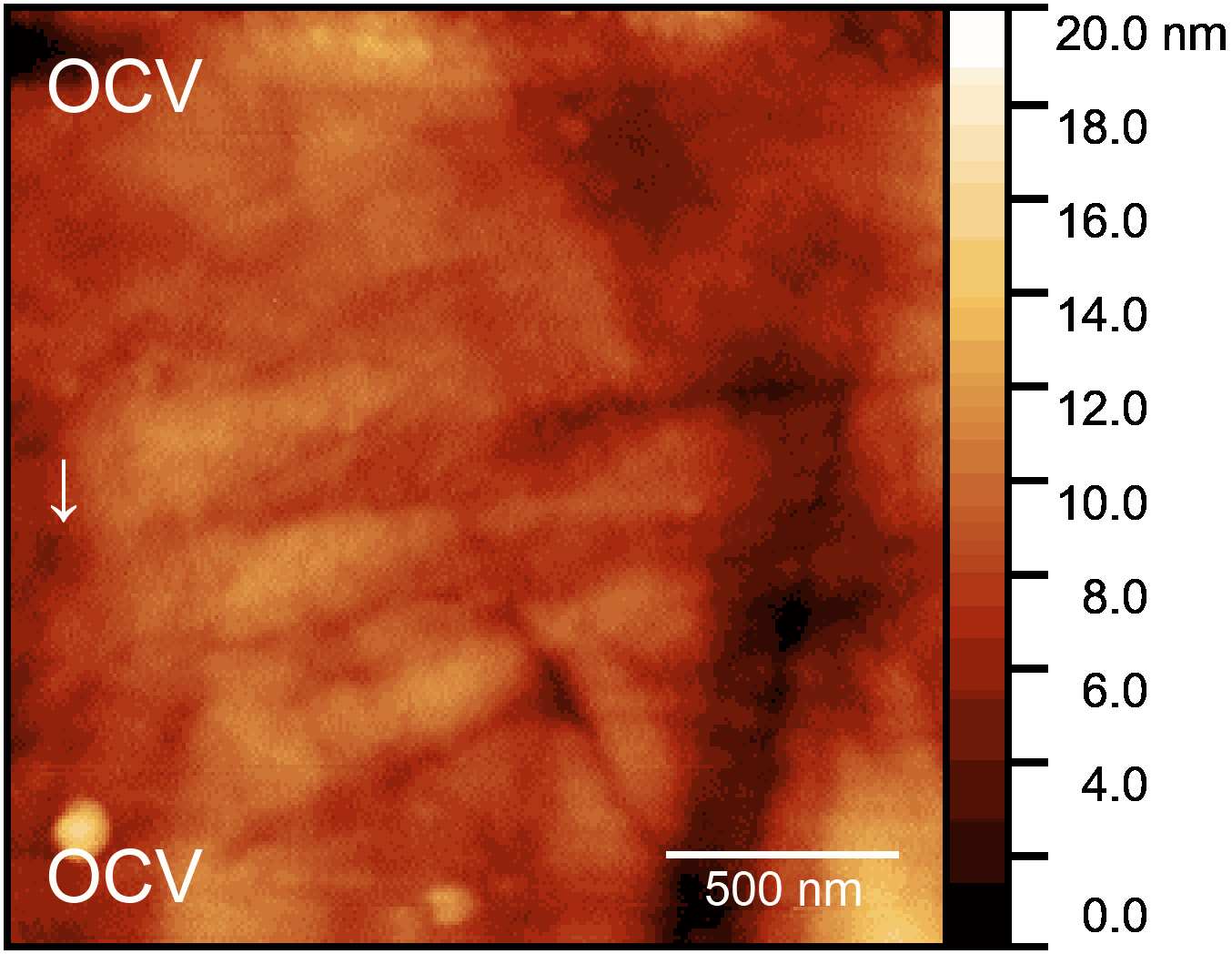


Supporting Media 1: *Operando* EC-AFM of SiN_0.40_ thin film


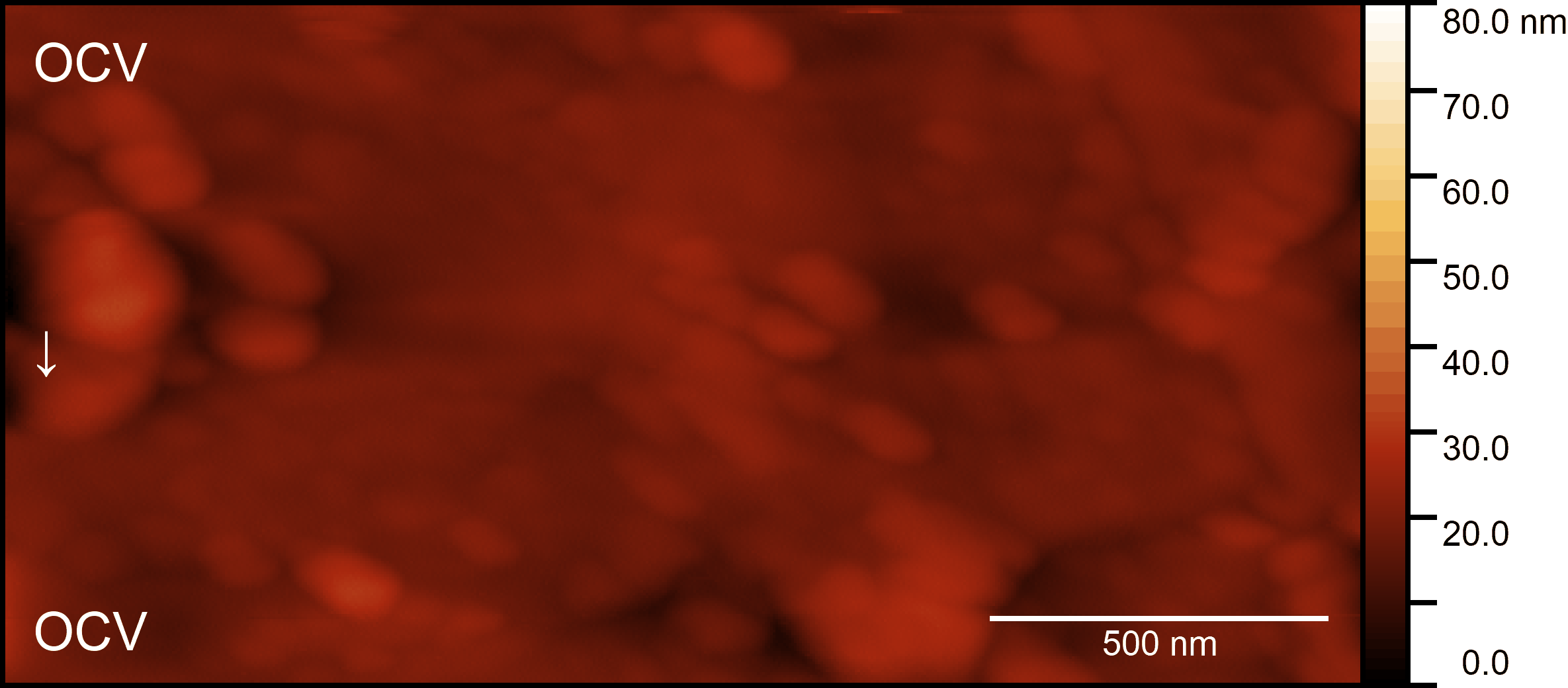


Supporting Media 2: *Operando* EC-AFM of Si thin film
